# Three-dimensional printing educational anatomical model of the patellar luxation in dogs

**DOI:** 10.1371/journal.pone.0255288

**Published:** 2021-07-30

**Authors:** Beatriz Ribeiro Gaspar, Antonio Chaves de Assis Neto

**Affiliations:** Department of Surgery, School of Veterinary Medicine and Animal Science, University of Sao Paulo, São Paulo, Brazil; Faculty of Veterinary Medicine - University of Lisbon, PORTUGAL

## Abstract

**Background:**

Few studies are available for assessing the current situation of 3D printing in veterinary medicine, due to the recent popularization of this technology. This study aimed to simulate a 3D model of the femorotibiopatellar joint of dogs based on the medial patellar luxation. The scanning, editing and printing of the femur, tibia, fibula and patella of a dog from the Laboratory of Anatomy of FMVZ USP were performed.

**Results:**

Three femorotibiopatellar joint models were printed: one representing a healthy join without alterations; the second one with the medially deviated tibial tuberosity; and a last one representing the shifted tibial tuberosity and the trochlear sulcus flattened as consequence. The 3D edition consisted of medial rotation of the tibia and tibial tuberosity (22° against the healthy tibia), and the flatten of the medial femoral condyle (0.2 cm) and femoral trochlear groove. After printing, the corresponding measurements were taken with the alterations and the bone models were made with elastics to represent the anatomical components of the dog joint. Finally, the measurements corresponding to the distance from the patellar ligament to the lateral femoral condyle were taken in each specimen, in order to observe the change in position of the ligament according to the occurrence of the bone alterations.

**Conclusion:**

We printed 3D articular anatomical components of the femurotibiopatellar joint that could be valuable educational tools for the study of medial patellar luxation in dogs.

## Introduction

Three-dimensional (3D) printing, is a technology that has been applied in human medicine in recent years [1–3]. Used as an alternative to the use of animals in education and research, this technology also allows the anticipated knowledge of the conformation and anatomy of an organ or region of anatomical-clinical interest [4–9]. The study of the knee of dogs, as well as other regions, can also benefits of this technology, since we can analyze the structures that compose it and how they interact mechanically.

The patellofemoral joint in the dog is formed by the articular face of the patella and the femur. The ligaments are divided into patellar retinaculars, patellofemoral and patellar ligaments. In carnivores, the patella attaches to the tuberosity of the tibia through a single patellar ligament, which is formed by the distal portion of the femoral quadriceps muscle insertion tendon. The patellar bone is the largest sesamoid in the animal body and is built-in within the tendons of the quadriceps muscle group. The patella has two functions: (1) to maintain stability in a straight line during the concentration of the quadriceps muscle group, and (2) to provide mechanical efficiency to this same muscle group [10].

According to Largon [11] the patella exerts multiple roles, and the principal one is function like an anatomical pulley for the extensor quadriceps muscle, acting as a driver of the knee extension forces throughout its movement. Additionally, the sesamoid exerts protection of the femoral trochlea, since it interposes between the quadriceps tendon and the femur, preventing the excessive friction between the tendon and the femoral condyles.

The patellar luxation is the loss of the normal anatomical relationship between the trochlear sulcus of the femur and the patella, be it congenital or traumatic. The dislocation can be medial, common in small breeds; lateral, more recurrent in large breeds or, less frequently, in both directions. The patellar luxation is ranked into four grades (G): G1, where patella dislocates manually during extension of the knee, but returns to the trochlea when released; G2, where the patella displaces more easily when manipulated and there is intermittent claudication; G3, where the patella is permanently dislocated, but can be reduced with manipulation. The femoral trochlea is shallow or even flat; and G4, where patellar dislocation is permanent, without any possibility of manual reduction. The patella is above the medial condyle and the trochlea is superficial, absent or even convex [12].

The musculoskeletal abnormalities are associated with medial patellar luxation, including *genu varum*, medial rotation and lateral bending of the distal third of the femur, medial displacement of quadriceps muscle group, distal femoral epiphysis dysplasia, rotational joint instability, degenerative joint disease, shallow trochlear groove with hypoplasic or absent medial border, and medial rotation of the tibia with medial deviation of its crest [13]. A widely accepted theory explains that the problem begins in the hip, with femoral varus and a decreased anteversion of the femoral epiphysis and diaphysis. This leads to a decrease in the angle of inclination between the femoral diaphysis and its longitudinal axis, associated with a smaller caudo-cranial angle of the femoral body. This abnormality in the growing animal displaces medialy the extensor muscles, leading to an impairment of the medial femoral growth associated with the accelerated growth of its lateral and distal extremities; the result is medial inclination and rotation of the distal epiphysis of the femur and the proximal epiphysis of the tibia [14].

Digital resources and 3D models have been proven indispensable tools for educational purposes, medical training, design reconstructive surgeries, surgical planning, orthopedic procedures, bioprintings and other clinical applications [15]. The usefulness of 3D printing in the anatomical knowledge of organs and body regions prove its importance in teaching and its advantage in ethical, cultural, logistic and financial aspects compared to traditional anatomical pieces [16].

In the simulation of patellar luxation we can choose to show some specific bone changes, and the study with printed 3D parts allows students and interested professionals to see how these changes modify the arrangement of some of the basic articular constituents of the knee, besides making it possible to know which structures are affected and how they alter the biomechanics of this joint.

## Materials and methods

### Anatomical technique procedure

The canine bones, belonging to the collection of the Veterinary Macroscopic Anatomy Laboratory of the School of Veterinary Medicine and Animal Sciences of the Universidade de São Paulo (FMVZ-USP), were used as templates for digitization (Approval of the Ethics Committee on the Use of Animals in Education: 4923110716). The animal was a small to medium-sized dog, random bred, with unknown weight and age, and without any orthopedic changes in the bones that make up the left femorotibiopatellar joint. The corpses were prepared according to standard procedures for osteotechnics; soft tissues were initially removed and posteriorly submitted to the maceration process by boiling water, and then cleaned and dried [17]. All the bones were deposited in a hydrogen peroxide solution (50%) to lighten the pieces for 12 hours. Subsequently, the bones were colored.

### Scanning and 3D editing of knee

The bones were digitalized using the "3D Go! SCAN^®^" model (Creaform Inc. Lévis, Quebec, Canada). This scanner has two HD digital cameras, each surrounded by a set of four white LED bulbs, and a projector that emits a white light pattern. The cameras detect the surface of an object and acquire images that are displayed by the software VXelements (*Lévis*, *Quebec*, *Canada*) in the form of a mesh composed of thousands or even millions of triangles. This software handles the acquisition and processing of 3D data resulting from 3D scanner digitalization. The images of the bones were formed in real time. The digitized images were edited using the software Meshmixer (Cary, NC, USA). This software allows for correcting formed images, using tools to exclude some uneven surfaces, flattening bumps possibly formed, to smoothen meshes, to reduce noise, and to fill flaws. The overall time needed for scanning was 30 minutes. Through this software it was possible to homogenize the images and perform some of the bone changes, such as medial displacement of the crest of the tibia—which in this example is the primary consequence of the femoral varus that leads to the medial patellar luxation—and the flatten of the trochlear groove and medial condyle, as the last alteration.

### 3D impression and wash

The digital files used to produce the bones (biomodels) were printed using the 3D printer-mojo^®^ (Rehovot, Israel). The printer uses the *Fused Deposition Modeling TM* (FDM) technology in the form of a thermoplastic filament-shaped material (acrylonitrile-butadiene-styrene, ABS) deposited together with support resin on a removable semi-adherent base 12.7 cm × 12.7 cm. The printing time for each piece varied from 10 to 11 hours. The models were produced in real scale (original size-100%) and in a more economical mode of printing. The cost for each bone was approximately US$ 2,50.

The printed parts were cleaned in a WaveWash 55 –Stratasys^®^ (Rehovot, Israel) washer to remove the support resin, thereby leaving only the part that was being manufactured. In the cleaning process, which lasted approximately 8 hours, the washer used a specific cleaning agent (Econoworks Tables Cleaning Agent^®^) that removed only the support resin. The printed models were infilled with ABS.

## Results

### 3D digital models and changes

**Fig 1:**
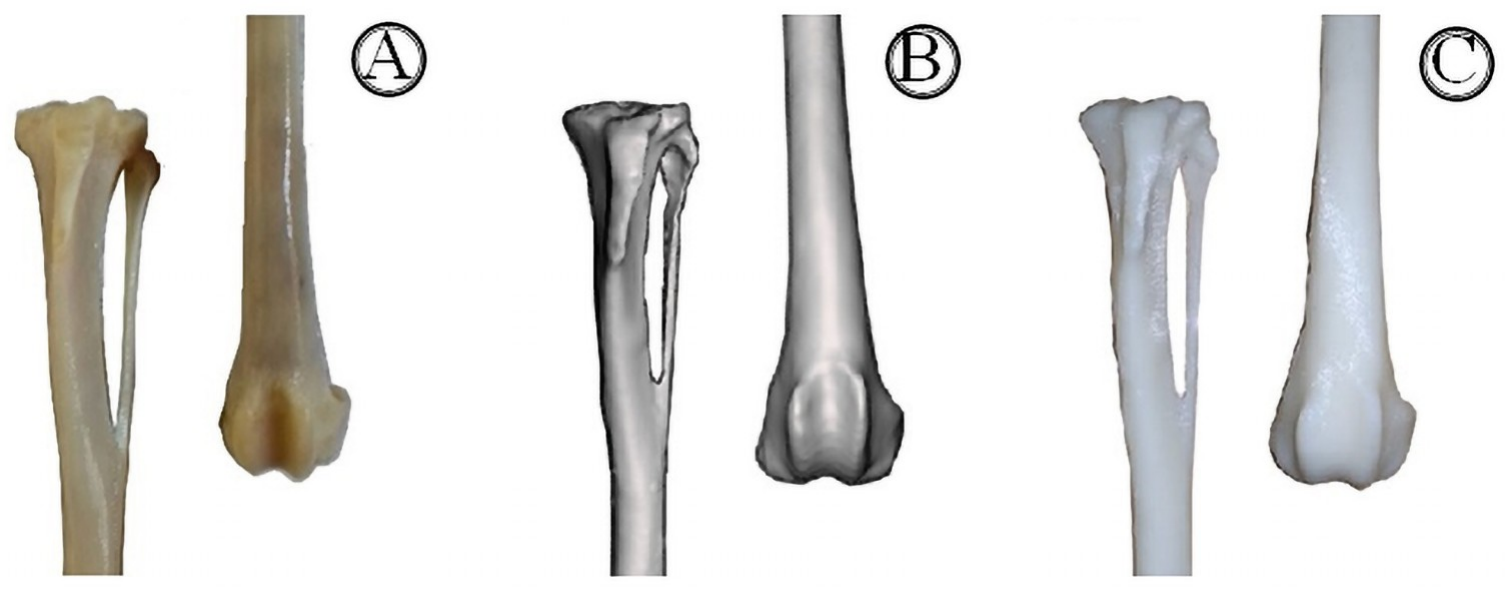
Healthy bones after maceration (A), edition (B) and printing (C), respectively. Focus on the proximal border of the tibia and the distal part of femur.

Three femorotibiopatellar joint models were printed: one representing a healthy join without alterations; the second one with the medially deviated tibial tuberosity; and a last one representing the shifted tibial tuberosity and the trochlear sulcus flattened as consequence. The specimens were printed on 2/3 of the bones (Fig 1) (proximal part of the tibia and distal part of the femur) and 74.2% of the original size, so that they fit the printing platform and had their focus turned to articular region. All six pieces were adjusted to the same size so that there was no incorrect fitting (Fig 1).

### Alterations in femoral trochlear sulcus and tibial tuberosity

**Fig 2:**
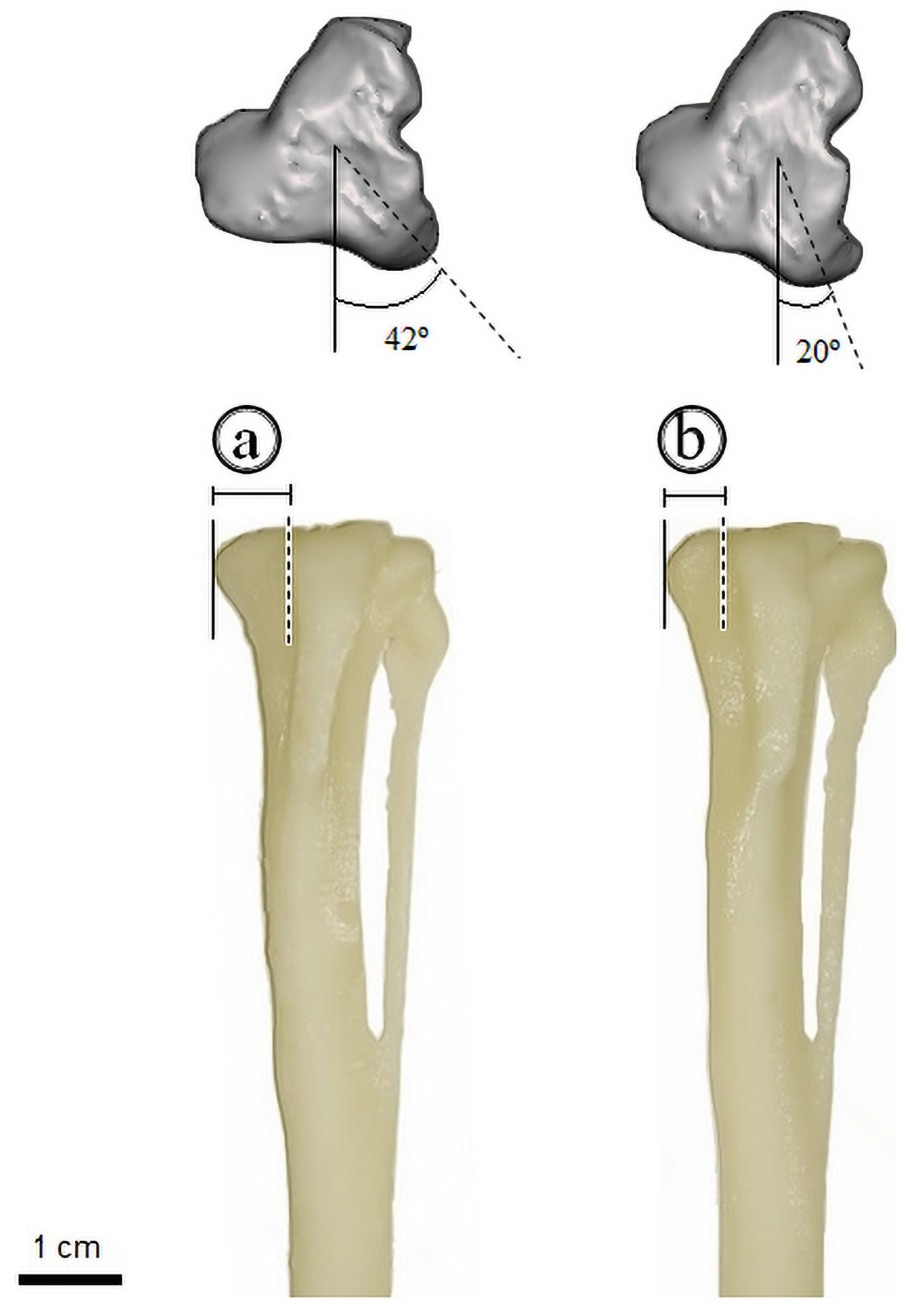
Difference between angulation and milimetry after the medial deviation of the tibial tuberosity. On the left is the healthy bone, in which the angulation of the tibial crest is 42° and the distance between its tuberosity and medial condyle is (a) = 0.75 cm. In the right is the bone with medial deviation, where the angulation is 20° and the distance turned (b) = 0.60 cm. The solid line is fixed, and the dotted line is movable.

**Fig 3:**
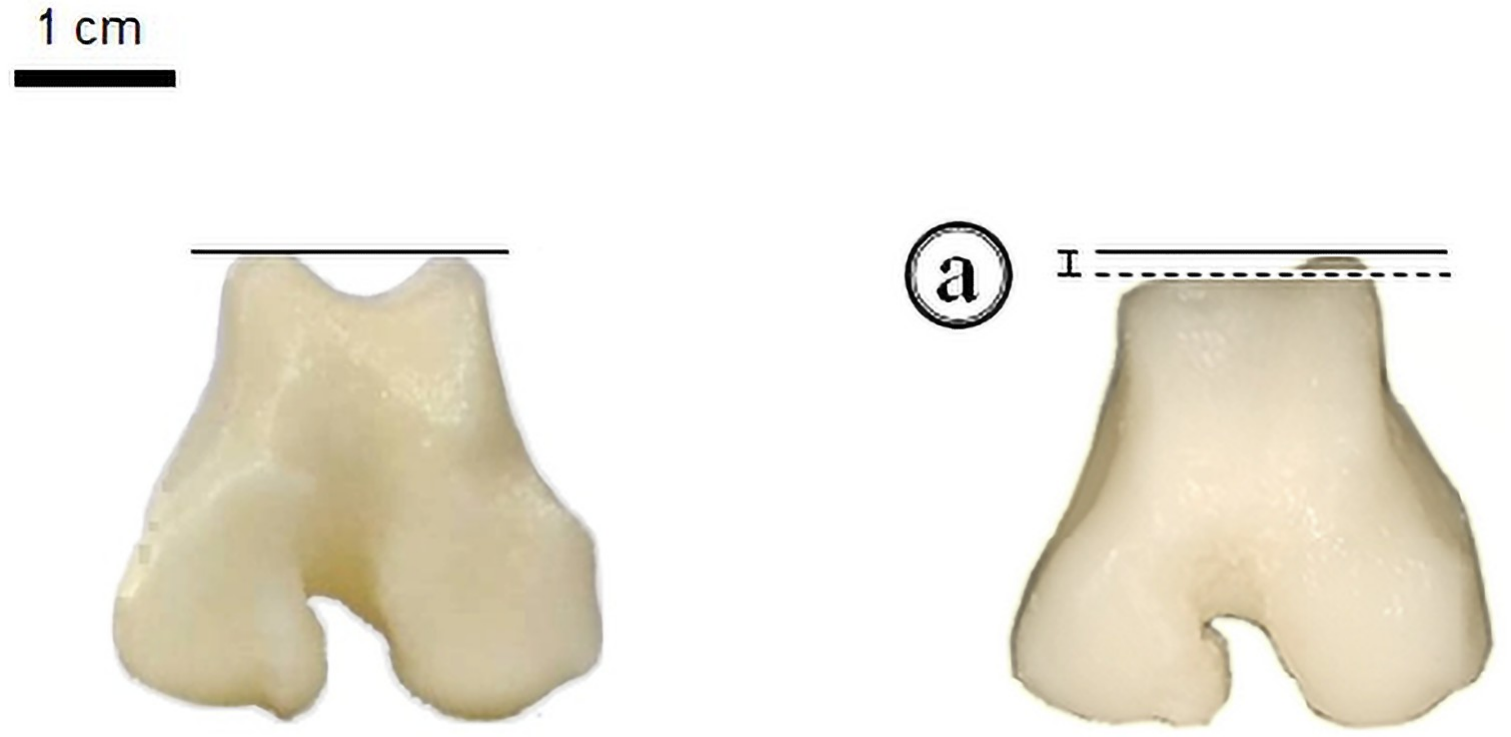
Height difference: (a) = 0.2 cm in the depth of the trochlear groove and medial condyle in the normal piece (left) and the edited one (right).

The tuberosity of the tibia was deviated medially, with a difference of a—b = 0.15 cm in the modified part in comparison to the tibia piece without alterations. In addition to that, a difference of 42° − 20° = 22° in the tibial tuberosity angle due to the medial deviation was obtained (Fig 2), considering a fixed point drawn from the center of the tibial epiphysis to its division with the medial condyle. In the femur, the trochlear groove and medial condyle were flattened to represent a difference of a = 0.2 cm against its initial positions in the healthy femur (Fig 3).

### 3D joints with ligaments

**Fig 4:**
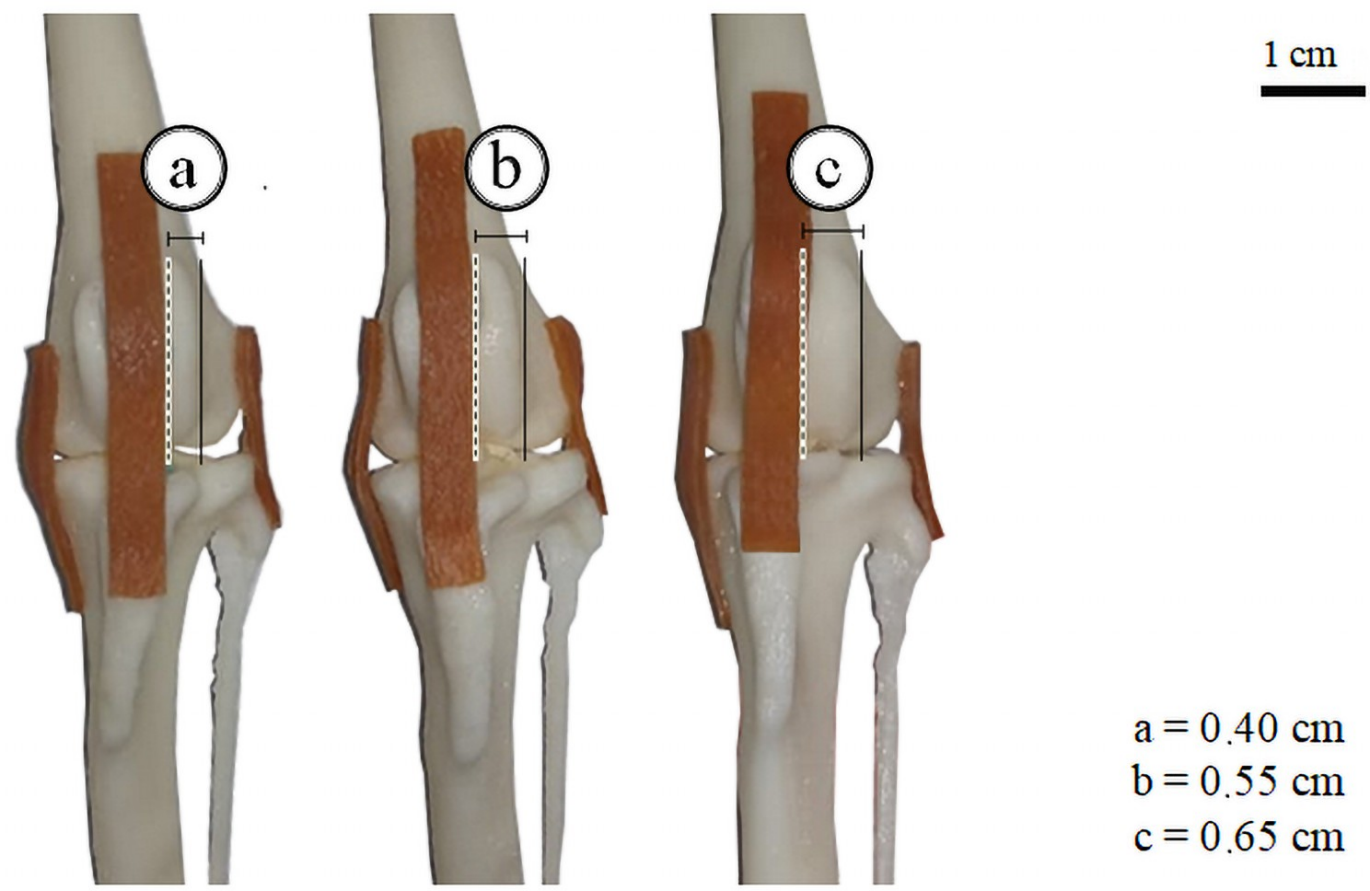
Three-stages of the femorotibiopatellar joint: Medial patellar luxation. (a) Healthy articulation, without morphological alterations; (b) Joint with medial rotation of tibia and its tuberosity; (c) Articulation with the rotated tibia associated with the flattening of trochlear sulcus and medial condyle. In the corner: variation of the distance as the moving line (dotted) moves away from the fixed line (continuous).

According to Piermattei et al. [12], in the patellar luxation grade two we can find the tibial crest rotated medially up to 30°. The medial deviation of the tuberosity in the modified bone, represented in Fig 4a, was 22 degrees. The Fig 4b shows the flattening of the trochlear groove as an outcome to the deviation of the tibial crest, caused by the absence of retropatellar pressure in the trochlea and patellar friction in the medial condyle, serving as a representation of the evolution of a grade 2 dislocation and pointing to the intrinsic relationship between patellar dislocation and trochlear dysplasia [18]. In both specimens of Fig 4b and 4c, it is possible to reduce the patella in the correct anatomical position, both by the digits and lateral rotation of the tibia, characterizing the severity of the dislocation in question.

It is possible to visualize that the medial rotation of the tibia and consequently of its tuberosity lead to a deviation of the patellar ligament, modifying the anatomical relationship of the articular components of the knee (Fig 4).

## Discussion

For the first time, 3D printed model of medial patella luxation in dog was demonstrate as potential educational application. 3D printing technology has been used in different field: ophthalmological, orthopedic, neurosurgery, and dentistry. However, in veterinary medicine, this technology still gaining popularity. Patellar luxation is classified as a developmental disorder, since the condition is not usually present at the moment of birth, but is a result of congenital bone, conformational and biomechanical changes, being diagnosed in the first stages of dog development [19]. There are also studies that affirm that the bone conformation of the femur and tibia is significantly different between dogs with the condition and healthy dogs, further strengthening the concept that the existence of bone alterations may be the origin or consequence of patellar dislocation [20].

Studies in veterinary medicine courses support the use of 3D models as complementary tools in education [9, 21–24]. In our study, the 3D model was able to simulate different grades of medial patella luxation. However, recent studies just analyzed three-dimensional kinematics of the patella in dogs during walk and trot [25] and, the influence of femoral varus deformities on patellar alignment using a computer model [26]. Physiologically, when the animal is growing, the patella is responsible for exerting retropatellar pressure in the femoral trochlea, leading to the development of a sulcus with depth and widths adequate for the correct slip of the patella. When this mechanism fails, the absence of pressure leads to the underdevelopment of the trochlear groove [19, 27], as represented in the printed pieces (Fig 3). The traction exerted by the patellar ligament on the medial condyle precedes its hypoplasia, which also contributes to the aggravation of the condition, since condylar hypoplasia is one of the factors favoring patellar luxation [28, 29]. The luxation, however, may be aggravated as early bone changes trigger subsequent changes.

There are several causes that can lead to the angular alteration responsible for the incorrect fit of the patella in the trochlear groove, and among those we can mention the tibial valgus, which may or may not occur as a consequence of the femoral varus. The rotational alteration of the tibia or tibial trochlea, medially, leads to displacement of the insertion site of the patellar ligament, contributing to medial patellar luxation [19]. The patellofemoral joint is complex, and representations patellar luxation in cadaveric and *in vivo* studies is difficult. Therefore, the 3D model presented here will support to overcome the limitation and difficulties faced in cadaveric or *in vivo* studies.

The didactic representation of patellar luxation can be made according to which initial process led to the disease. In the case of 3D printing it is possible to simulate the bone changes that lead to it, making possible anatomical-clinical and biomechanical learning of the condition, besides allowing the study of different approaches to correct it. In the case of lesions, this technology allows a deeper knowledge of the surgical area, allowing a more precise repair and reconstruction of the injured tissue [30].

The creation of medial patella luxation from 3D model could be an excellent way to obtaining didactic models for teaching veterinary medicine. In a previous study, we showed that the cost is relatively cheap [6]. 3D printing is increasingly becoming a tool that not only complements the traditional methods of anatomical studies, but also promises to be extremely useful for the development of new techniques and approaches in medicine [31].

## Conclusion

The 3D printing was able to simulate the different degrees of medial patella luxition in the dog. The proposed 3D printed model can be a valuable educational tool for the study of medial patellar luxation in dog. The articular anatomical components, as well as small angular changes can views on the models 3D printed. This will be important to allow planning and training of surgical procedures.

## Supporting information

S1 File (XLSX)
